# 1104. Comparison of Antibiotic Sampling Techniques: Predicting Plasma Vancomycin Concentrations Using Volumetric Absorptive Microsampling (VAMS) from Capillary and Venous/Arterial Whole Blood

**DOI:** 10.1093/ofid/ofab466.1298

**Published:** 2021-12-04

**Authors:** Kevin J Downes, Derrick Tam, Anna Sharova, Christina Vedar, Julie C Fitzgerald, Abbas F Jawad, Ganesh S Moorthy, Athena F Zuppa

**Affiliations:** Children’s Hospital of Philadelphia, Philadelphia, Pennsylvania

## Abstract

**Background:**

Therapeutic drug monitoring (TDM) is paramount to optimize the safety and efficacy of vancomycin (VAN). In children, TDM is challenged by difficulty in obtaining venous samples, impeding timely sampling. We assessed the ability of volumetric absorptive microsampling (VAMS) as a novel, whole blood sampling technique to predict plasma VAN concentrations in plasma.

**Methods:**

We conducted a prospective pilot study among critically ill children prescribed VAN for clinical care. Coincident with VAN TDM in plasma (P), we collected 20 µL of capillary whole blood (C) and venous/arterial whole blood (V) using VAMS. Paired VAMS-P samples drawn >5 mins apart and VAMS samples with over- or under-loaded filter tip on visual inspection were excluded. Plasma concentrations were measured via chemiluminescent immunoassay in the Chemistry Laboratory. VAMS C and V concentrations were measured using LC/MS in the Bioanalytic Core Laboratory. Plasma concentrations were predicted from whole blood VAMS with Passing-Bablok regression using 3 methods: 1) uncorrected VAMS measures, 2) hematocrit-corrected VAMS, and 3) lab-corrected VAMS (Figure 1). We then assessed bias, imprecision, and accuracy of plasma predictions from VAMS (C and V) as compared to coincident P concentrations for each technique (Figure 1).

Figure 1. Methods for relating whole blood vancomycin concentrations collected via VAMS to plasma concentrations and measure to evaluate predictive performance.

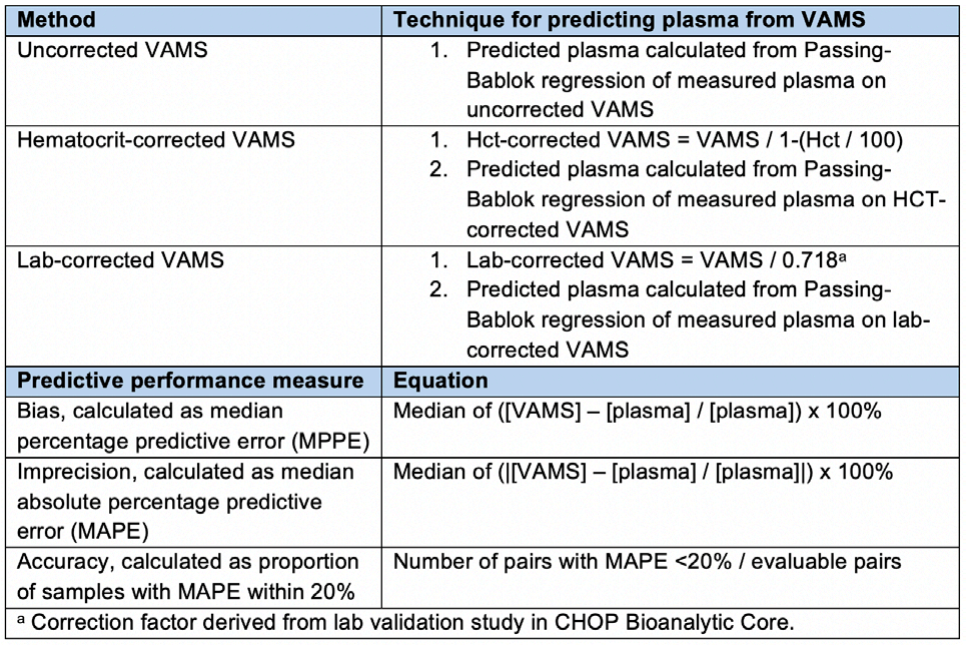

**Results:**

Paired samples were collected from 31 enrolled subjects (Figure 2), with a median age of 3.3 years (range 0.1-17.9). Measured P concentrations ranged from 4.6 - 54.9 mg/L. 11 C samples (29%) and 3 V samples (10%) were excluded due to collection issues. Prediction results are shown in Figure 3. The 3 prediction techniques had similar performance characteristics, with each method displaying minimal bias (-0.4-2.0%) and reasonable imprecision (13.7-20.2%). The accuracy of prediction of P concentrations using VAMS was better for V than C samples.

Figure 2. Flow diagram from sample collection to evaluation.

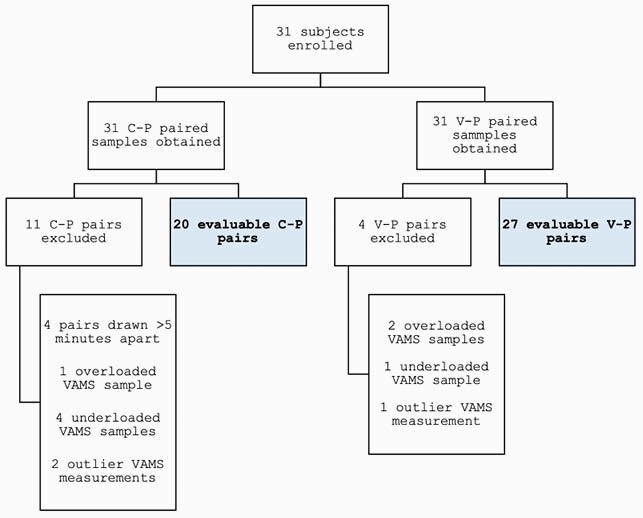

Abbreviations: C-P, capillary VAMS-plasma; V-P, venous/arterial VAMS-plasma; VAMS, volumetric absorptive microsampling.

Figure 3. Performance of 3 techniques to predict plasma vancomycin concentrations using whole blood collected via VAMS.

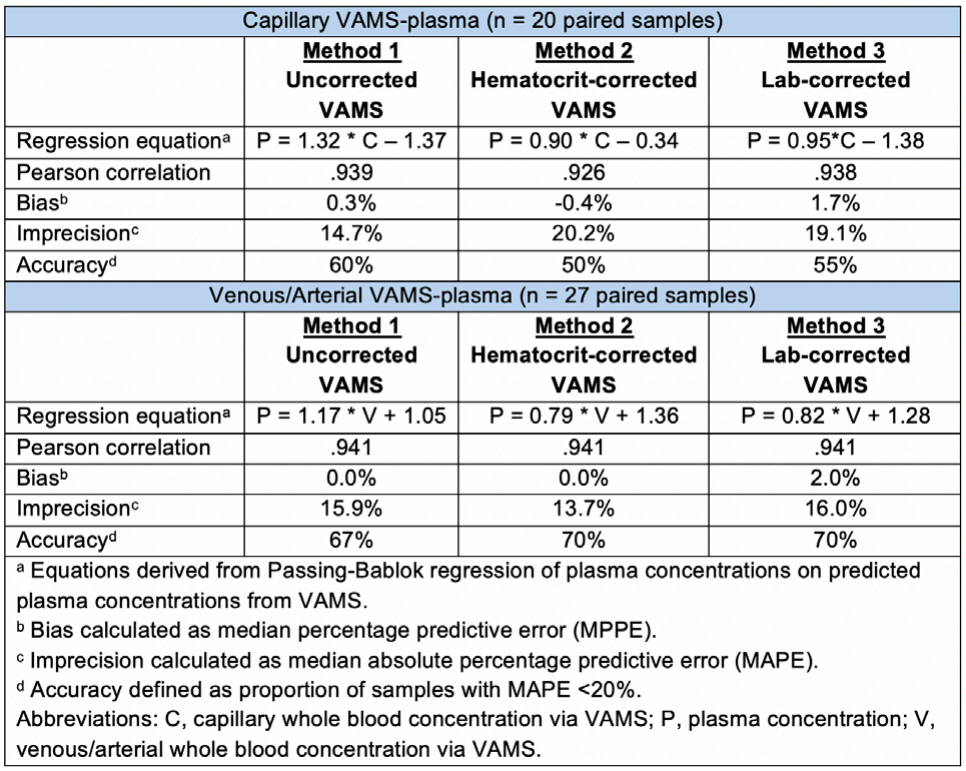

**Conclusion:**

Our pilot highlights the challenges of using VAMS for TDM. Sample collection issues were common. When VAMS is used, education on collection techniques is imperative. The predictive performance of VAMS was modest and V sampling had higher accuracy than C, although our sample size was small. Larger studies will be needed to further evaluate the predictive performance of the regression equations derived by our study.

**Disclosures:**

**Kevin J. Downes, MD**, **Merck, Inc.** (Grant/Research Support)

